# Cannabinoids and triple-negative breast cancer treatment

**DOI:** 10.3389/fimmu.2024.1386548

**Published:** 2024-08-08

**Authors:** Luka Dobovišek, Simona Borštnar, Nataša Debeljak, Simona Kranjc Brezar

**Affiliations:** ^1^ Division of Medical Oncology, Institute of Oncology Ljubljana, Ljubljana, Slovenia; ^2^ Medical Centre for Molecular Biology, Institute of Biochemistry and Molecular Genetics, Faculty of Medicine, University of Ljubljana, Ljubljana, Slovenia; ^3^ Department of Experimental Oncology, Institute of Oncology Ljubljana, Ljubljana, Slovenia; ^4^ Faculty of Medicine, University of Ljubljana, Ljubljana, Slovenia

**Keywords:** triple-negative breast cancer, cannabinoids, endocannabinoid system, chemotherapy, immunotherapy, immune system, tumor microenvironment, immune checkpoint inhibitors

## Abstract

Triple-negative breast cancer (TNBC) accounts for about 10-20% of all breast cancer cases and is associated with an unfavorable prognosis. Until recently, treatment options for TNBC were limited to chemotherapy. A new successful systemic treatment is immunotherapy with immune checkpoint inhibitors, but new tumor-specific biomarkers are needed to improve patient outcomes. Cannabinoids show antitumor activity in most preclinical studies in TNBC models and do not appear to have adverse effects on chemotherapy. Clinical data are needed to evaluate efficacy and safety in humans. Importantly, the endocannabinoid system is linked to the immune system and immunosuppression. Therefore, cannabinoid receptors could be a potential biomarker for immune checkpoint inhibitor therapy or a novel mechanism to reverse resistance to immunotherapy. In this article, we provide an overview of the currently available information on how cannabinoids may influence standard therapy in TNBC.

## 1 Triple-negative breast cancer

Triple-negative breast cancer (TNBC) accounts for about 10-20% of all breast cancer cases and is associated with the worst prognosis. TNBC is a heterogeneous group of tumors defined by the absence of the estrogen receptor (ER), the progesterone receptor (PR), and the absence of the human epidermal growth factor receptor 2 (HER2). It mainly occurs in younger patients and patients with BRCA germline mutations (1, 2). TNBC has been divided into six different subgroups: basal-like 1 (BL1), basal-like 2 (BL2), mesenchymal (M), mesenchymal stem-like (MSL), immunomodulatory (IM) and luminal androgen receptor (LAR) (3). The TNBC microenvironment (TME) includes tumor-infiltrating lymphocytes (TILs) with CD3+ T and CD20+ B lymphocytes, CD38+/CD138+ plasma cells, tumor-associated macrophages (TAMs), cancer-associated fibroblasts (CAFs), tumor-associated neutrophils (TANs), natural killer (NK) cells and cancer-associated adipocytes (CAAs). The TNBC TME is unique in that it has a high expression of vascular endothelial growth factors and a high infiltration rate of TILs and TAM and plays an important role in the pathogenesis of the disease. Type M2 macrophages are upregulated compared to other subtypes of breast cancer. CAAs also play a greater role. A variety of cytokines and chemokines influence tumor growth, metastasis and drug resistance, mediate immunosuppression and antitumor activity, and play an important role in TNBC TME (4). Dendritic cells (DCs) are antigen-presenting cells (APCs) that play a crucial role in acquired and innate immune responses and are involved in the development of T cell-mediated antitumor immune responses (5). Neoangiogenesis is necessary for the nutrition and oxygen supply of the tumor and thus for the progression of the tumor. The endothelium is involved in the invasion of tumor cells into the vascular lumen and the resulting metastasis. In addition, endothelial cells are one of the main sources of CAFs (6). Until recently, treatment options for TNBC were limited to chemotherapy, e.g. anthracyclines and taxanes. The prognosis of metastatic TNBC is poor, with a median survival of 11 to 17 months. A new successful systemic treatment is immunotherapy with inhibitors of programmed cell death ligand 1 (PD-L1) and programmed cell death protein 1 (PD-1), also known as immune checkpoint inhibitors (ICI). Atezolizumab (PD-L1 inhibitor) and pembrolizumab (PD-1 inhibitor) are in clinical use (7). Atezolizumab is used for the treatment of metastatic or unresectable locally advanced breast cancer and is restricted to patients whose tumors express PD-L1 on immune cells (> 1% PD-L1 positive tumor cells) (8). Pembrolizumab is used in early-stage TNBC (9) and in patients with advanced cancer with a combined positive score (CPS score), defined as the number of PD-L1–staining cells (tumor cells, lymphocytes and macrophages) divided by the total number of viable tumor cells multiplied by 100) > 10 (10). In TNBC PD-1/PD-L1 are used as biomarkers for response to immunotherapy. However, new tumor-specific biomarkers are needed to improve patient outcomes (11). In patients with BRCA mutation, poly (ADP-ribose) polymerase (PARP) inhibitors are used in adjuvant and metastatic treatment. This is an example of synthetic lethality and targeted treatment (12, 13). Newer treatment options are the antibody-drug conjugates sacituzumab govitecan and trastuzumab deruxtecan. Sacituzumab govitecan is an antibody directed against Trop2 to which SN38 (a derivative of irinotecan) is bound (14). Trastuzumab deruxtecan is an antibody-drug conjugate consisting of the humanized monoclonal antibody trastuzumab covalently linked to the topoisomerase I inhibitor deruxtecan (a derivative of exatecan). It is used in a subset of TNBC that is considered HER2-low, a category that blurs the distinction between HER2-positive and TNBC (15).

## 2 Cannabinoids and the endocannabinoid system

Cannabinoid receptors (CBRs) are membrane-bound G protein-coupled receptors (GPCRs) and were identified over 30 years ago. Cannabinoid receptor 1 (CB1R) is one of the most abundant receptors in the brain. To a lesser extent, CB1R is also expressed in the periphery, including the immune system. Cannabinoid receptor 2 (CB2R) is also expressed in the central nervous system, but to a much lesser extent than CB1R. In addition, CB2R is highly expressed in the periphery, particularly in organs that are part of the immune system and in other peripheral tissues. Natural polymorphisms and alternative splice variants may be important for its function (16). In addition, there is a “non-canonical” extended signaling network of the endocannabinoid system. It consists of fatty acid derivatives, ionotropic cannabinoid receptors (transient receptor potential (TRP) channels) and other GPCRs (GPR18, GPR19, GPR55, peroxisome proliferator-activated receptor alpha (PPARα), eCB), enzymes involved in the biosynthesis and degradation of endocannabinoids (fatty-acid amide hydrolase 1 (FAAH) and monoacylglycerol lipase (MAGL)), and protein transporters fatty-acid-binding proteins (FABPs)) (17–22). Cannabinoid receptors recognize different agonists and antagonists and can be activated by endogenous or exogenous cannabinoids. Endogenous cannabinoids or endocannabinoids are metabolites of arachidonic acid (AA). The best studied are N-arachidonoylethanolamine (anandamide, the Sanskrit word for “bliss”) and 2-arachidonoylglycerol (2-AG), which together with the CBRs form the endocannabinoid system (23). Anandamide binds to CBRs with higher affinity than 2-AG, but acts only as a partial agonist (24). 2-AG, on the other hand, behaves as a full agonist for CBRs (25, 26). 2-AG binds selectively to CBRs, whereas anandamide is less selective (27). It has been suggested that 2-AG and not anandamide is the actual natural ligand for CBRs (28–30). Tetrahydrocannabinol (THC) is the main psychoactive constituent of *Cannabis sativa* and thus an exogenous phytocannabinoid and a non-selective agonist of CB1R and CB2R (31). Other important phytocannabinoids are cannabidiol (CBD), cannabichromene (CBC), cannabigerol (CBG), cannabidivarin (CBDV) and tetrahydrocannabivarin (THCV) (32). Compared to THC, CBD has a lower CB1R and CB2R affinity and acts as an inverse agonist on CB2R. It is a non-psychoactive substance and therefore a potential therapeutic agent (33). Synthetic cannabinoids (i.e. JWH-015, WIN55,212-2, compound-10, etc.) are a heterogeneous group of substances that can be selective agonists or antagonists of CB1R or CB2R (34). CB1R and CB2R exhibit allosteric binding and biased signaling that influences the biological response mediated by agonists (31, 35).

## 3 Cannabinoids and the immune system

Various components of the endocannabinoid system act as important regulators of the immune system and the immune response (36–38). Exogenous cannabinoids are generally immunosuppressive and potent immunological mediators (39–43). Immune cells express both CB1R and CB2R (44, 45), although the expression of CB1R is significantly lower compared to CB2R (41, 46). CB2R is mainly found in cells of the immune system and plays an important role as a modulator of immune function (47–49). The activation of CB2R in many immune cells is central to the suppressive effects of cannabinoids. However, the pro-inflammatory effects appear to be linked to the expression of CB1R. Cannabinoids inhibit adenylyl cyclase and activate beta-gamma-mediated signaling pathways and modulate intracellular free calcium levels. These changes may negatively affect the release of inflammatory mediators and the induction of pro-inflammatory transcriptional programs. Exposure to cannabinoids inhibits the release of prostaglandins, histamine, and matrix-active proteases from mast cells (50). Phagocytic function is suppressed by cannabinoids. Cannabinoids also suppress inflammation at a secondary level by downregulating the production of cytokines (e.g. tumor necrosis factor alpha (TNFα), interferon gamma (IFNγ), interleukin-1 (IL-1), and IL-4 (41, 51–54). There are relatively few descriptions of immunological side effects of cannabinoids in humans. However, the human immune system is altered by chronic exposure to cannabinoids (55). Cannabinoid exposure does not accelerate the loss of immunocompetence in HIV-1 infected patients (56, 57). Cannabis use is associated with long-term changes in immunological homeostasis (58, 59). Inhibition of cell-mediated immunity has been found in marijuana smokers (60) and cannabis smoking causes inflammatory changes in the airways (61). Studies have shown that exposure to cannabinoids leads to a suppression of responsiveness to infectious diseases, and a link between cannabis use and increased susceptibility to various infections has been suggested (62–64). These studies suggest a significant potential for immunological side effects of cannabinoid compounds in humans. Interestingly, cannabinoids can affect the gut microbiota (65–67), which also influences immunotherapy for cancer (68–71).

## 4 Cells of the TNBC microenvironment and the endocannabinoid system

It appears that most of the cell types present in the TNBC TME express CBRs and may be affected in some way by the exogenous cannabinoids and/or the endocannabinoid system (4, 72). CB2R is expressed in CD8+ T lymphocytes and CD4+ T lymphocytes and CB1R mRNA transcripts are modestly present in human T- lymphocytes (40, 41, 73). The expression of the CB2R receptor is low in circulating T lymphocytes. However, several studies have reported that CB2R receptor expression is increased in activated T lymphocytes and that its activation decreases their proliferation (74–76). This is associated with reduced production of pro-inflammatory cytokines and increased apoptosis (74–78). In addition, CBR agonists can upregulate immunosuppressive cytokines (79, 80) and cause inhibition of chemotaxis (81–83). The antitumor function of T lymphocytes is enhanced in *Cnr2* conditional knockout mouse (84). The activation of CB2R appears to have different effects depending on the subtype of T lymphocytes, with the functions of Th1 and Th17 tending to be reduced and those of Th2 promoted in humans (72, 75, 85, 86). In addition, the CB2R receptor is highly expressed in cytokine-induced killer (CIK) cells, a subset of cytotoxic T lymphocytes with a CD3+ CD56+ immunophenotype (4, 87–90). It is important to note that the activation of T lymphocytes is the primary mechanism of action of ICI (91, 92). B-cells express CB1R and CB2R (93, 94). Treatment with a CB2R agonist increased the proliferation of B lymphocytes, a phenomenon that was blocked by a CB2R antagonist (95). In mice, activation of the CB2R receptor was associated with differentiation, migration, proliferation and antibody class switching of B lymphocytes (96–98). These results suggest that CB2R is part of the immune programming of B lymphocytes and plays an important role in the development of B lymphocytes (72, 99). CBRs are overexpressed in chronic lymphocytic leukemia (CLL) compared to healthy B lymphocytes and CBR1 could be a new prognostic marker (100). CB2R is expressed in monocytes and CB1R mRNA transcripts are modestly present (40, 41, 73). CB2R agonists can modulate human monocyte migration (101, 102). Macrophages express both CB1R and CB2R (103, 104). Several studies have shown that cannabinoids negatively regulate phagocytosis, cell-spreading, and antigen presentation by macrophages (105–107). CB2R has been shown to switch the polarization of M1 macrophages to M2 macrophages (108–110). CB2R is expressed in human fibroblasts (111) and transcripts for CB1R and CB2R have been found in both odontoblasts and gingival fibroblasts (112). Fibroblasts also express enzymes that metabolize endocannabinoids. Fibroblasts can be influenced by autocrine signaling of endocannabinoids via CB1R and CB2R and paracrine signaling by neighboring leukocytes (113). CB2R is expressed in polymorphonuclear neutrophils (40, 41, 73). Cannabinoids can influence neutrophil function via CBRs or other mechanisms. The inhibitory effect of cannabinoids on neutrophil functional responses is mostly related to a mechanism other than CBRs, consistent with the absence or very low expression of CB2R (114–119). Neutrophil migration is related to the expression of CB2R and GPR55 (120). Chemotaxis of human neutrophils is inhibited by CBR agonists (114, 121). CB1R and CB2R are expressed in NK cells. The predominance of CB2R in NK cells is illustrated by a striking ratio of 100:1 between CB2R and CB1R (40, 41, 44, 73, 122–125). NK cells release large amounts of endocannabinoids (125) and endocannabinoids influence the chemotaxis of NK cells (126). NK cell activity is inhibited by CBR agonists (127), and cannabis use has been associated with a decrease in NK cell counts (58, 59). Mature adipocytes from visceral and subcutaneous adipose tissue express CBRs on their plasma membranes and CB1R is located at various subcellular levels, including the plasma membrane and mitochondria of the adipocyte (128, 129). However, there is some uncertainty regarding CB2R expression in differentiated adipocytes (130–134). A complete endocannabinoid system has been found in both murine and human adipocytes (135–137). Bone marrow-derived DCs from mice express CB1R and CB2R (138) and the endocannabinoid system is present in human DCs (139). Endocannabinoids act as chemoattractants for DCs and activation of CBRs induces apoptosis (138, 140). Both CB1R and CB2R are expressed in endothelial cells (141, 142). It is possible that an atypical cannabinoid receptor, the endothelial cannabinoid receptor (eCB receptor), is responsible for the vasodilatory effect of cannabinoids (143). A study found that CBRs are expressed in glioblastoma endothelial cells. CB1R expression was detected in about 38% and CB2R expression in 54% of cells. Compared to CB1R, the expression of CB2R was increased in the endothelial cells of glioblastoma (142).

## 5 Cannabinoid receptor expression in TNBC

The presence of CBRs in human breast tumors was investigated by quantitative real-time PCR and confocal microscopy. Lower levels of CB1R mRNA were detected in low, intermediate and high histologic grade tumors compared to normal, non-cancerous breast tissue. In all tumors examined, CB2R expression was higher than CB1R expression. Hormone receptor-negative (HR-) tumors expressed more CB2R mRNA than ER +/PR + tumors (144). Perez-Gomez et al. performed a histopathologic analysis of tissue samples for the expression of CBRs. A very large proportion of human breast adenocarcinomas (~75%) expressed CB2R and expression was strongly associated with HER2+ tumors, while no association was found between CB2R expression and HR+ or TNBC. There was an association between increased expression of CB2R and poorer prognosis and higher likelihood of local recurrence in HER2+ breast cancer (145). The orphan receptor GRP55 could play an important role in TNBC. In the study by Andradas et al. it was found that the TNBC cell line MDA-MB-231 reduced its invasive behavior when GPR55 expression was knocked down. Similar effects were observed in an animal model of lung metastasis. In breast cancer patients, there is a strong association between GPR55 protein levels and TNBC tumors. Using tissue microarrays and publicly available data from The Cancer Genome Atlas (TCGA), it has been shown that higher GPR55 expression is associated with poorer patient prognosis (lower disease-free survival and metastasis-free survival) (146).

## 6 Effect of different cannabinoids on TNBC

The antitumor effect of THC on breast cancer cell lines was documented. Among the tumor cells, those with a more aggressive phenotype, including the MDA-MB-231 cell line, were more sensitive to THC. One mechanism of this effect is that THC arrests the cells at the G_2_-M cell cycle checkpoint via downregulation of the cyclin-dependent kinase 1 (Cdc2), as suggested by the reduced sensitivity of Cdc2-overexpressing cells to THC (144). In addition, CBD has an antitumor and anti-metastatic effect in TNBC cell lines. The antitumor effect of CBD is mediated by the activation of apoptosis. Apoptosis in the MDA-MB-231 cell line can be induced by direct or indirect activation of the CB2R receptor and transient receptor potential vanilloid type-1 (TRPV1) as well as by an increase in intracellular Ca^2+^ and reactive oxygen species (ROS) independent of the cannabinoid/vanilloid receptors (147). CBD induces an interaction between the PPARγ, the mammalian target of rapamycin (mTOR) and cyclin D1, which promotes apoptosis (148) and can downregulate the expression of an inhibitor of basic helix-loop-helix transcription factors (Id-1) in metastatic TNBC cells, which leads to a reduction in tumor aggressiveness (149). The downregulation of Id-1 expression is the result of differential modulation of extracellular signal-regulated kinase (ERK) and ROS. In addition, the pro-differentiation factor Id-2 is upregulated by CBD. The anti-metastatic activity was also confirmed in animal models, in which CBD significantly reduced the primary tumor mass as well as the size and number of metastases in the lung (150). The study by Elbaz et al. showed that CBD inhibits the growth and metastasis of breast cancer cells by inhibiting epidermal growth factor (EGF)/EGFR signaling and modulating the tumor microenvironment, and that it can significantly inhibit EGF-induced proliferation and chemotaxis of breast cancer cells. Inhibition of EGF-induced activation of the EGFR, ERK, protein kinase B (AKT) and nuclear factor kappa B (NF-κB) signaling pathways and the secretion of the matrix metallopeptidases (MMP2 and MMP9) has been described. In *in vivo* models, the analysis of the molecular mechanism showed that CBD significantly inhibits the recruitment of TAMs in the primary tumor stroma and in secondary lung metastases (151). The selective CB2R agonist JWH-015 reduced the primary tumor burden and metastasis of the luciferase-labeled murine TNBC 4T1 cell line in immunocompetent mice *in vivo* and reduced the viability of murine 4T1 cells *in vitro* by inducing apoptosis. The reduction in cell viability mediated by JWH-015 was not dependent on Gαi signaling *in vitro*, nor was it altered by classical pharmacological blockade of other CBRs (CB1R, GPR55, TRPV1). The effect of JWH-015 was calcium-dependent and led to changes in mitogen-activated protein kinase (MAPK)/ERK signaling (152). Rimonabant (SR141716), a CB1R antagonist, inhibits the proliferation of the TNBC cell line MDA-MB-231 more effectively than ER+ cell lines. It also shows an antiproliferative effect *in vivo* by reducing the volume of xenograft tumors induced by injection of MDA-MB-231 in mice. Rimonabant inhibits the growth of human TNBC cells via a CB1R-lipid raft/caveolae-mediated mechanism (153). A strong synergy between rimonabant and erastin in inhibiting the growth of TNBC cells has been described both *in vitro* and *in vivo*. This occurred by increasing the levels of lipid peroxides, malondialdehyde (MDA), 4-hydroxynonenal (4-HNE) and ROS production in the cytosol, enhancing intracellular glutathione (GSH) depletion and inducing G_1_ cell cycle arrest (154). Cannabidiolic acid activates the expression of PPARβ/δ target genes in the MDA-MB-231 cell line (155). The molecular interaction between the cannabinoid agonists of vetiver oil and the CBR2 receptor was found to be the cause of the cytotoxicity of vetiver oil on the 4T1 cell line (156). Styrene-maleic acid nanomicelles encapsulating WIN55,212-2 were synthesized to reduce the side effects and increase the efficacy of the drug. The synthetic cannabinoid analog WIN55,212-2 is a synthetic CBR agonist with a cytotoxic effect on the MDA-MB-231 cell line, but causes psychoactive side effects (157). The synthesis and evaluation of a selective, non-psychotropic CB2R agonist, designated compound 10, with *in vivo* activity against MDA-MB-231 cells was reported. This novel cannabinoid o-quinone (compound 10) has been described to induce cell apoptosis through CB2R activation and oxidative stress. Importantly, compound 10 showed no toxic effects on non-cancerous human mammary epithelial cells or *in vivo* (158). Photodynamic therapy for TNBC was combined with CB2R agonists for TNBC. Synergistic effects were observed in both *in vitro* (MDA-MB-231 cell line) and *in vivo* experiments. The survival time of tumor-bearing animals was significantly prolonged (159).

## 7 Cannabinoids and triple-negative breast cancer treatment

### 7.1 Cannabinoids and chemotherapy in TNBC

A brief overview of the research on cannabinoids and chemotherapy in TNBC is summarised in **Table 1**. CB2R expression is increased in the MDA‐MB‐231 cell line when treated with chemotherapy. Overexpression of CB2R had an inhibitory effect on TNBC cells and significantly improved their sensitivity to chemotherapy with cisplatin, doxorubicin and docetaxel. Therefore, CB2R may be involved in the chemosensitivity of the MDA‐MB‐231 cell line to chemotherapy (160). The antitumor efficacy of pure THC was compared with that of an herbal drug preparation of fresh cannabis flowers containing a variety of cannabinoids and terpenes. The herbal drug preparation contained THC and CBG, but no CBD, and was more effective than pure THC in producing antitumor responses in cell cultures and animal models of various breast cancer subtypes, including the TNBC subtype (MDA-MB-231 and SUM159 cell lines). In the study by Blasco-Benito et al. different combinations of chemotherapy with THC and herbal preparations were tested. The combination of THC or the herbal preparation with paclitaxel or epirubicin had no effect on the decrease in cell viability. When the herbal preparation was added to cisplatin treatment, an enhanced antiproliferative response was observed. Furthermore, this was tested *in vivo* by injecting the MDA-MB-231 cell line into immunodeficient mice. The herbal drug preparation was significantly more potent than pure cannabinoid (the same dose of THC was administered). The combination of the herbal drug preparation with cisplatin had neither a positive nor a negative effect on the antitumor effect of platinum chemotherapy (161).

**Table 1:** *In vitro* and *in vivo* studies investigating the effect of cannabinoids on TNBC chemotherapy.

Anti-cancer drug	Cannabinoid	Cell line	Animal model	Result (*in vitro*)	Results (*in vivo*)	Study
Cisplatin, doxorubicin, Docetaxel	Not available	MDA-MB-231	Not available	Increased CB2R expression. Increased sensitivity to antitumor drugs cells overexpressing CB.	Not available	(160) Song Q et al
Paclitaxel Epirubicin	THC, herbal drug preparation of fresh cannabis flowers (THC, CBG).	MDA-MB-231 SUM159	MDA-MB-231 cell line into immunodeficient female nude mice	The combination of THC or the herbal preparation with paclitaxel or epirubicin had no effect on the decrease in cell viability. Herbal preparation added to cisplatin produced enhanced antiproliferative response.	The herbal drug preparation was more effective than pure cannabinoid. The combination of the herbal drug preparation with cisplatin had neither a positive nor a negative effect on the antitumor effect of platinum chemotherapy.	(161) Blasco-Benito S et al
Doxorubicin	nano-micellar formulation of the synthetic cannabinoid WIN55,212-2	4T1 MDA-MB-231	Female Balb/c mice implanted with 4T1 mammary carcinoma	Decreased the cell viability	Cannabinoid nano-micelles were able to reduce the psychotropic effect of WIN55,212-2. The combination of doxorubicin and cannabinoid nano-micelles potentiated the antitumor effect of both drugs and reduced tumor volume.	(162) Greish K et al
Cisplatin	CBD	MDA-MB-231	Not available	The addition of CBD did not attenuate the effect of cisplatin.	Not available	(163) Dobovišek L et al
Doxorubicin	CBD	MDA-MB-231 MDA-MB-468	Not available	Increased sensitivity to doxorubicin.	Not available	(164) Surapaneni SK et al
Doxorubicin	CBD-loaded extracellular vesicles	MDA-MB-231	MDA-MB-231 xenograft model of athymic nude mice	CBD-loaded extracellular vesicles alone or in combination with doxorubicin reduced inflammation and metastasis and promoted apoptosis.	The combination of extracellular CBD vesicles and doxorubicin significantly reduced tumor burden.	(165) Patel N et al
Cisplatin	CBD	MDA-MB-231	Not available	Not available	No antiproliferative effect at low CBD doses. At intermediate doses CBD exerts an antagonistic effect on treatment with cisplatin. CBD exerts a strong cytotoxic effect at high doses.	(166) D’Aloia A et al

Greish et al. investigated the effects of a nano-micellar formulation of the synthetic cannabinoid WIN55,212-2, a CBR agonist, on TNBC. Cannabinoid nano-micelles decreased the cell viability of TNBC cell lines (4T1 and MDA-MB-231). In addition, the antitumor effect of cannabinoid nano-micelles was investigated in mice with 4T1 tumors. Cannabinoid nano-micelles were more effective compared to free WIN55,212-2 and were able to reduce the psychotropic effect of WIN55,212-2. In addition, administration of cannabinoid nano-micelles sensitized the 4T1 cell line to the effect of doxorubicin. This effect was also observed *in vivo*. The combination of doxorubicin and cannabinoid nano-micelles potentiated the antitumor effect of both drugs and reduced tumor volume (162). The addition of CBD did not attenuate the effect of cisplatin in the MDA-MB-231 cell line (163). In combination with CBD, increased sensitivity to doxorubicin was observed in MDA-MB-468 cells. This was partly due to activation of apoptosis and inhibition of autophagy. The combination of CBD and doxorubicin decreased lysyl oxidase (LOX) and integrin-α5 and increased caspase-9 protein in the MDA-MB-468 cell line (164). CBD-loaded extracellular vesicles isolated from human umbilical cord mesenchymal stem cells and encapsulated by sonication with CBD sensitize TNBC cells to doxorubicin in both *in vitro* and *in vivo* models. CBD-loaded extracellular vesicles alone or in combination with doxorubicin reduced inflammation and metastasis and promoted apoptosis in the MDA-MB-231 cell line through cell cycle arrest in G1 phase and downregulation of IL-17, NF-κB, Twist, phosphorylated transducer and activator of transcription-3 (pSTAT3), STAT3 proteins *in vitro*. This was confirmed by *in vivo* studies, which showed that the combination of extracellular CBD vesicles and doxorubicin significantly reduced tumor burden. The combination modulated the tumor microenvironment by decreasing the expression of transforming growth factor-beta (TGFβ), IL-6, NF-κB, Integrin α−5 (ITGA5), Smad-2, GPC 1&6 and Twist and mediated apoptosis by increasing the expression of Bcl-2-associated X protein (BAX) and caspase 9 and decreasing the expression of Bcl2. The study concluded that extracellular CBD vesicles increase the sensitivity of the MDA-MB-231 cell line to doxorubicin, thereby reducing the required effective dose of doxorubicin and thus reducing or eliminating toxicity, and that extracellular vesicles can be used as potential delivery systems for cannabinoids due to their easy internalization by tumors (165). One study found that a threshold mechanism rather than a dose-dependent curve better describes the CBD effects on the MDA-MB-231 cell line. The threshold is reached between 3 and 5 μM. At low doses, CBD exerts no antiproliferative effect. At intermediate doses, near the threshold concentration, CBD induces survival mechanisms, including cell cycle arrest and autophagy, and exerts an antagonistic effect on treatment with cisplatin. In contrast, at high doses (> 5 µM), CBD exerts a strong cytotoxic effect by activating bubbling death (166). Importantly, the major cannabinoids (THC, CBD, and cannabinol) and their metabolites found in the plasma of cannabis users can inhibit several P450 enzymes, including CYP2B6, CYP2C9, and CYP2D6, and cause pharmacokinetic interactions between these cannabinoids and xenobiotics that are extensively metabolized by these enzymes (167). There is evidence that cannabinoids alleviate peripheral neuropathic pain caused by chemotherapy (168–170) and prevent doxorubicin-induced cardiomyopathy (171–173). Both are side effects of taxane and anthracycline chemotherapy, which is frequently used in TNBC (174, 175).

### 7.2 Cannabinoids and Immunotherapy

Exposure to THC can promote growth and metastasis of the 4T1 cell line. Specifically, THC exposure leads to an accelerated onset of detectable tumors, larger tumor sizes, and a higher number of lung metastases. The suppression of the immune response against the 4T1 tumor by THC may be responsible for the increased tumor growth and metastasis. The study by McKallip et al. showed that MDA-MB-231 expresses low levels of CB1R and undetectable levels of CB2R. Therefore, it was hypothesized that the degree of tumor sensitivity to THC is directly related to the degree of CB1R and CB2R expression and that THC may only kill tumors that express CBRs. However, THC exposure may lead to increased growth and metastasis of tumors with little or no expression of CBRs due to suppression of the antitumor immune response (176). Two studies investigated the effects of cannabis use during immunotherapy with ICI. A clinical retrospective analysis including 140 patients with advanced melanoma, non-small cell lung cancer and clear cell renal carcinoma showed a worse response rate (RR) in cannabis users (37.5% RR with nivolumab alone versus 15.9% in the nivolumab-cannabis group). Cannabis use was not a significant factor for progression-free survival (PFS) or overall survival (OS) (177). A prospective observational study by Bar-Sela et al. included 102 patients with advanced cancers (melanoma, non-small cell lung cancer and clear cell renal carcinoma) treated with ICI (anti-PD1 and anti-PDL1). Cannabis use correlated with a significant reduction in time to tumor progression and OS. Cannabis users were associated with a lower number of immune-related adverse events (iAEs). Blood samples from patients taken before the start of treatment were analyzed for endocannabinoids and endocannabinoid-like substances. A single endocannabinoid-like lipid, 2-oleoyl-glycerol (2-OG), was associated with significantly different levels between groups. The concentrations of AA, 2-AG, N-docsatetraenoyl ethanolamide (DtEA), linolenic acid (LnA), N-linoleoyl ethanolamide (LEA), N-oleoyl amide (O-Am), N-oleoyl ethanolamide (OEA), N-palmitoyl ethanolamide (PEA) and N-stearoyl ethanolamide (SEA) were significantly affected by the onset of immunotherapy. The levels of O-Am and OEA increased, while the levels of the other substances decreased after immunotherapy. Four lipids (measured before immunotherapy) correlated with OS: increased levels of N-arachidonoylamide (A-Am) were associated with better OS expectancy, and an inverse relationship was observed between OS and SEA, 2-AG and 2-linoleoylglycerol (2-LG) (178). Another possible interaction between cannabinoids and ICI therapy is the gut microbiota, as already mentioned in this article (65–69).

### 7.3 Cannabinoids and other therapies in triple-negative breast cancer

There are no data on cannabinoids interfering with PARPi therapy and antibody-drug conjugates (sacituzumab govitecan and trastuzumab deruxtecan). However, it should be remembered that liver metabolism can be affected by cannabinoids, as mentioned above. PARPi olaparib is subject to extensive hepatic metabolism (mainly by the isoenzymes cytochrome P450 3A4/5 (179). Exatecan is primarily metabolized in the liver by hepatic P450 metabolism (CYP3A4 and CYP1A2) (180) and SN38 is metabolized in the liver by UDP-glucuronosyltransferase (UGT) (UGT1A1 and UGHT1A9) and UGT1A9 and other non-hepatic UGT enzymes (181). The main cannabinoids and their metabolites found in the plasma of cannabis users inhibit several P450 enzymes. THC competitively inhibits CYP1A2 and CBD competitively inhibits CYP3A4. THC-COOH is a substrate for UGT (167).

## 8 Discussion

**Figure 1:**
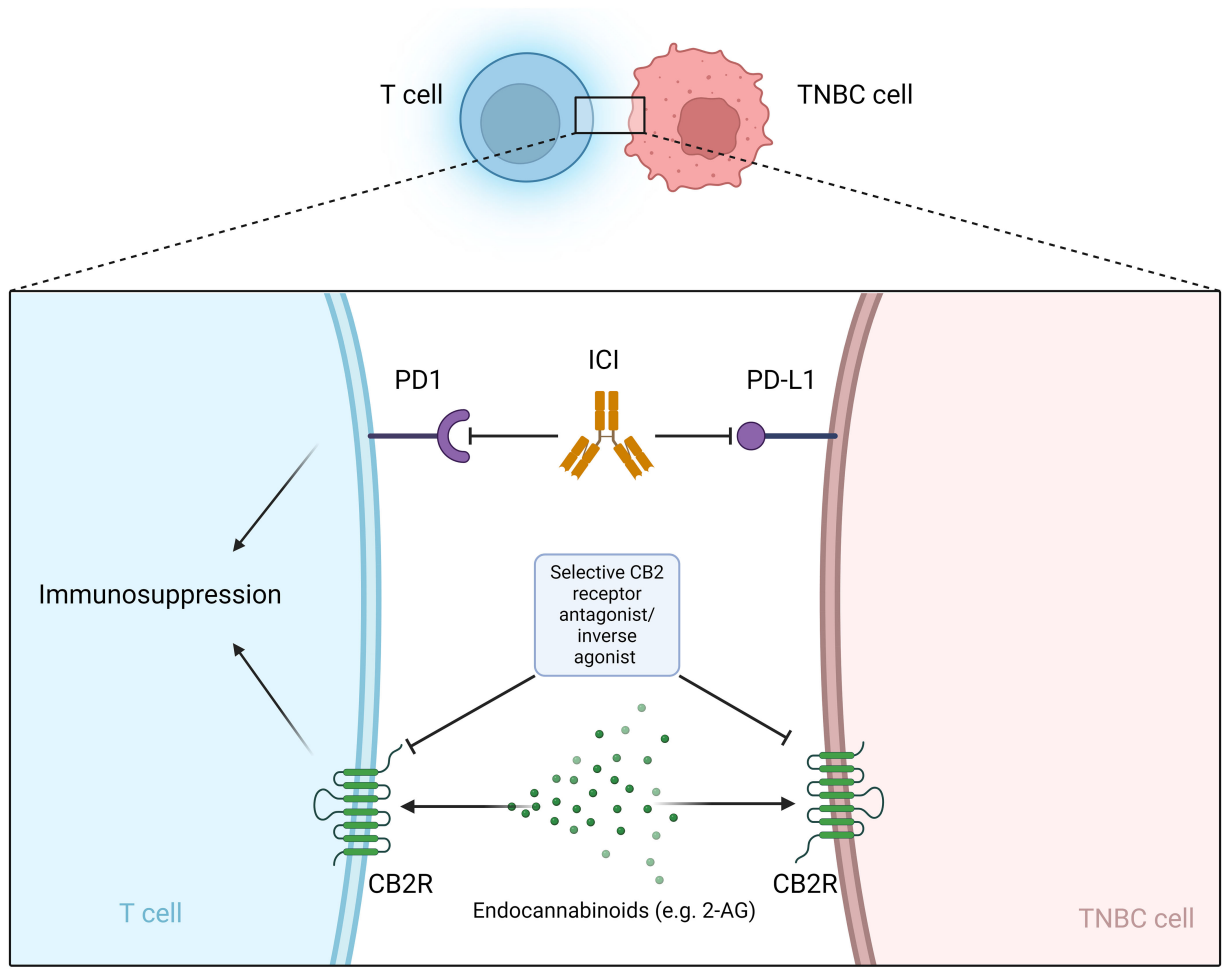
Selective CB2R antagonist/inverse agonist as a potential sensitizer for immunotherapy with ICI. ICI, Immune checkpoint inhibitors; PD-L1, programmed cell death ligand 1; PD-1, programmed cell death protein 1; CB2R, cannabinoid receptor 2; 2-AG, 2-arachidonoylglycerol.

Cannabinoids are already being used in palliative care for cancer patients (182). However, the panel of the Multinational Association of Supportive Care in Cancer (MASCC) advises against the use of cannabinoids as an adjuvant analgesic for cancer pain (183). In addition to breast cancer, cannabinoids influence tumor progression in various types of cancer, e.g. glioma/glioblastoma, skin, liver, colon, prostate (184–186). TNBC TME could be influenced by cannabinoids, however the role of CBRs on different cells infiltrating the TME is largely unknown (187). Preclinical and clinical data show that cannabinoids can help with chemotherapy-induced neuropathy (167–170). In addition, there is a worldwide trend toward legalization (e.g., in the United States and Canada) of recreational and medical use of cannabis and cannabis products, with new health concerns arising related to the increasing use of cannabis (188). Exposure to cannabis and the cannabinoids (THC, CBD and CBG) may be a risk factor for the development of breast cancer in the female population (189, 190). Therefore, many breast cancer patients are exposed to cannabinoids in one way or another during and/or after their oncologic treatment, but the safety of this exposure is uncertain and requires further research. Many patients take cannabinoids in the belief that this will help cure their disease, although there is currently no clinical data to support this claim in breast cancer patients, including TNBC. A survey of breast cancer patients found that 42% of survey participants used cannabis to treat symptoms and about half of these participants believed that cannabis could treat the cancer itself. Most participants used cannabis during active cancer treatment (191). There are no clinical data on whether cannabinoids interfere with standard TNBC treatment, although initial clinical trials show that patients with metastatic non-breast cancer who use cannabis while receiving ICI immunotherapy have poorer outcomes. The ability of cannabinoids to shorten the survival of patients treated with immunotherapy suggests the efficacy of cannabinoids and the potentially important role of the endocannabinoid system in generating the immune response triggered by ICI (177, 178). At the preclinical level, there is increasing evidence that the endocannabinoid system may play a role in tumor growth, treatment resistance, and metastasis. In most preclinical studies, various cannabinoids inhibited breast cancer cell lines and improved cancer outcomes in TNBC animal models (148–150, 192–194) usually without interfering with standard treatment (e.g., cisplatin, epirubicin, doxorubicin, paclitaxel) (162–165). However, tumor-promoting effects of cannabinoids have also been reported. An antagonistic effect of CBD on TNBC cell lines treated with cisplatin at intermediate doses was observed (166). THC exposure has been shown to stimulate the growth of the ER+ MCF-7 cell line (195) and can lead to increased growth and metastasis of tumors with little or no expression of cannabinoid receptors due to suppression of the antitumor immune response (176). Importantly, cannabinoids have immunosuppressive properties (39–43). Immunosuppression in the periphery occurs mainly through the modulation of CB2R, while CB1R are less involved in this process (44–46). Due to the current limitations of immunotherapy (non-response, disease progression and hyperprogression), new strategies are needed to sensitize cancer cells to immunotherapy (196) and modulators of CBRs may provide a new mechanism to achieve this goal (**Figure 1**). CBRs could be a predictive biomarker for ICI therapy or a novel mechanism to reverse resistance to ICI therapy. Importantly, phytocannabinoids are not selective for CBRs and therefore give us little insight into the role of CBRs in the pathophysiology of cancer. Selective CB2R agonists and antagonists are needed to develop potential anti-cancer drugs that target the endocannabinoid system (197).

## Author contributions

LD: Writing – original draft, Visualization, Validation, Supervision, Investigation, Conceptualization. SB: Writing – review & editing, Validation. ND: Writing – review & editing, Validation. SKB: Writing – review & editing, Visualization, Validation, Supervision, Conceptualization.
